# Multi-Organ Denervation: The Past, Present and Future

**DOI:** 10.3390/jcm14082746

**Published:** 2025-04-16

**Authors:** Syedah Aleena Haider, Ruth Sharif, Faisal Sharif

**Affiliations:** 1 Department of Medicine, University of Galway, H91 TK33 Galway, Ireland; 2 Department of Cardiology, University Hospital Galway, H91 YR71 Galway, Ireland; ruth.mcloughlin@universityofgalway.ie

**Keywords:** autonomic nervous system, sympathetic nervous system, hypertension, cardiometabolic syndrome, sympathetic denervation, endovascular denervation

## Abstract

The sympathetic division of the autonomic nervous system plays a crucial role in maintaining homeostasis, but its overactivity is implicated in various pathological conditions, including hypertension, hyperglycaemia, heart failure, and rheumatoid arthritis. Traditional pharmacotherapies often face limitations such as side effects and poor patient adherence, thus prompting the exploration of device-based multi-organ denervation as a therapeutic strategy. Crucially, this procedure can potentially offer therapeutic benefits throughout the 24 h circadian cycle, described as an “always-on” effect independent of medication compliance and pharmacokinetics. In this comprehensive review, we evaluate the evidence behind targeted multi-organ sympathetic denervation by considering the anatomy and function of the autonomic nervous system, examining the evidence linking sympathetic nervous system overactivity to various cardiometabolic and inflammatory conditions and exploring denervation studies within the literature. So far, renal denervation, developed in 2010, has shown promise in reducing blood pressure and may have broader applications for conditions including arrhythmias, glucose metabolism disorders, heart failure, chronic kidney disease and obstructive sleep apnoea. We review the existing literature surrounding the denervation of other organ systems including the hepatic and splenic arteries, as well as the pulmonary artery and carotid body, which may provide additional physiological benefits and enhance therapeutic effects if carried out simultaneously. Furthermore, we highlight the challenges and future directions for implementing multi-organ sympathetic ablation, emphasising the need for further clinical trials to establish optimal procedural technique, efficacy and safety.

## 1. Introduction

Recognised for its mediation of the ‘fight or flight’ response, the sympathetic division of the autonomic nervous system (ANS) plays a crucial role in maintaining homeostasis [1]. However, chronic overactivation of the sympathetic nervous system (SNS) has been implicated in a number of pathological processes. Beyond its established contribution to hypertension (HTN), these processes also contribute to the development and progression of numerous disease conditions, such as cardiometabolic syndrome, a triad of HTN, hypergylcaemia and dyslipidaemia [2].

In this comprehensive review, we describe the evidence behind SNS over-activation and provide an overview of the ANS, while considering the evidence supporting sympathetic denervation in three main organs (kidneys, liver and spleen), as well as denervation of the pulmonary artery and carotid body. We evaluate the potential for multi-organ neuromodulation and outline the challenges that may arise in its implementation.

## 2. Evidence for SNS Over-Activation

**Figure 1:**
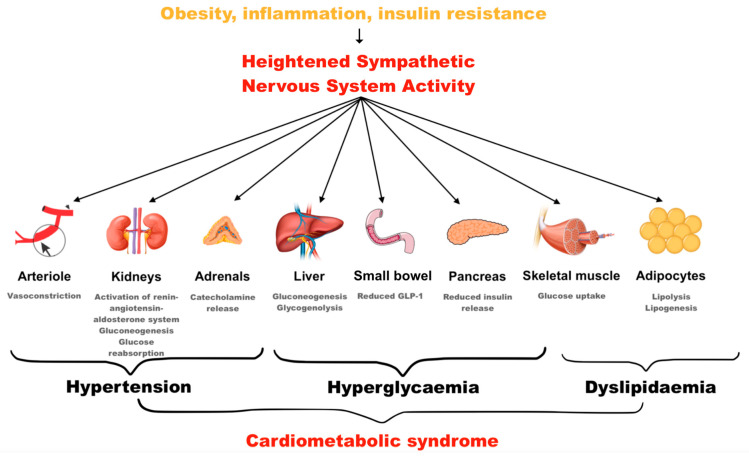
Manifestation of cardiometabolic syndrome via the sympathetic nervous system.

There is strong evidence linking SNS to many disease states [2]. Heightened SNS activity can precipitate a cascade of metabolic effects involving the renal endocrine system, culminating in complications such as endothelial dysfunction, insulin resistance and cardiac hypertrophy. Ultimately, this leads to cardiometabolic syndrome and obesity (Figure 1) [3]. In this state, SNS hyperactivity is further accelerated by visceral fat itself, due to excessive adipokine release [4]. Such a dangerous positive feedback loop is exacerbated by a disruption of the circadian rhythm in metabolic disorders, with the loss of nocturnal decline in blood pressure (BP) contributing to a heightened risk of end organ damage [5].

Another condition, obstructive sleep apnoea, is accompanied by a continuously elevated sympathetic drive even during resting normoxic wakefulness, independent of obesity [6,7,8]. Additionally, heart failure and pulmonary arterial HTN are characterised by an initial up-regulation of the SNS which eventually becomes maladaptive, with studies indicating a correlation between disease severity and the degree of sympathetic activation (measured by plasma norepinephrine levels and muscle nerve activity, respectively) [9,10,11]. In fact, epidemiological studies have noted the overlap of these disorders with the SNS at the centre. For example, the prevalence of cardiometabolic syndrome was 58% in patients with HTN and as high as 78% in patients with type 2 diabetes mellitus (T2DM), suggesting that this subpopulation of patients is more likely to respond to a sympathetic denervation therapy [12,13,14].

Further evidence indicating the significance of SNS control comes from immunological studies. The ANS controls both innate and adaptive immunity through its sympathetic and parasympathetic branches. Disruptions in this system can lead to changes in the inflammatory response, often seen in chronic autoimmune disorders such as systemic lupus erythematosus, systemic sclerosis and rheumatoid arthritis. The degree of SNS activation is closely associated with the level of inflammation and, interestingly, with cardiovascular risk [15].

In parallel, studies examining the benefits of parasympathetic nervous system (PNS) stimulation also offer support for the potential advantages of dampening SNS activity. For instance, vagal nerve stimulation techniques have recently emerged as a promising approach to mitigate ANS dysfunction, particularly in chronic conditions characterised by pain, chronic inflammation and reduced quality of life [16,17,18].

## 3. Neuromodulation: An Introduction

Understanding the effects of the SNS can guide the development of effective therapies for various medical conditions. Despite the sheer burden and frequent co-existence of such diseases, medication side effects and patient non-adherence limit the efficacy of pharmacotherapy [19,20,21]. For example, cardiometabolic syndrome represents a largely unmet therapeutic target with increasing costs and mortality and a global prevalence ranging from 12.5 to 31.4% [22]. In this context, the development of broad-based interventions such as multi-organ sympathetic ablation may represent a viable complementary intervention to curb the large-scale impact of chronic SNS overactivity and lessen the well-documented issues associated with medication compliance. Crucially, such a procedure can potentially attenuate sympathetic activity throughout the 24 h circadian cycle, described as an “always-on” effect independent of medical compliance and pharmacokinetics [23].

Of course, device-based therapy has evolved since its earlier surgical predicate. Dating as far back as the 1930s, the first series of surgical sympathectomies to manage HTN rendered significant side-effects and mortality rates [24]. Such procedures were deemed non-viable and only in 2010 did the focus shift to minimally invasive renal denervation (RDN), which had fewer complications and faster turnaround times [25]. While RDN interventions have garnered significant interest, an interventional procedure aimed at addressing sympathetic overactivity in multiple organs has the potential to be more potent and address a broader spectrum of conditions (see Table 1).

## 4. Autonomic Nervous System: An Overview

Understanding the anatomy of the ANS is vital for establishing SNS denervation procedures. The ANS is a component of the peripheral nervous system that regulates involuntary physiologic processes including heart rate, respiration, BP, digestion and sexual arousal [2]. It contains three anatomically separate divisions: the SNS, which activates the “fight or flight” response, the PNS, which activates the “rest and digest” state, and the enteric nervous system (ENS), which chiefly regulates digestion [41].

The sympathetic and parasympathetic nervous systems contain both afferent and efferent fibres, which contribute sensory input and motor output, respectively, to the central nervous system (CNS). Afferent fibres are responsible for numerous reflex activities that are processed at a subconscious level to produce visceral and/or somatic efferent (motor) responses. Generally, the SNS and PNS motor pathways consist of a two-neuron series: a preganglionic neuron with a cell body in the CNS and a postganglionic neuron with a cell body in the periphery that innervates target tissues [42].

The ENS comprises the myenteric (Auerbach) and submucosal (Meissner) plexuses. The myenteric plexus lies between the gastrointestinal tract’s longitudinal and circular muscles, coordinating peristalsis, while the submucosal plexus in the submucosa regulates water and electrolyte movement. Although the ENS functions independently through local reflexes, it interacts with the SNS and parasympathetic systems via postganglionic sympathetic or preganglionic parasympathetic neurons [41].

### Renal Innervation

Autonomic renal innervation, comprising sympathetic and parasympathetic components, forms the renal plexus. Sympathetic fibres originate from the aorticorenal, inferior mesenteric and superior mesenteric ganglia, while parasympathetic fibres arise from the vagus nerve and intermesenteric plexus (S2–S4). Both afferent and efferent nerves are present, though efferent pathways are exclusively sympathetic [43].

Renal nerves follow three paths: along the renal artery, diverging before entering it or joining its branches after the first bifurcation [44,45]. In the renal pelvis, afferent and efferent fibres separate but intertwine within the same bundle. Afferent nerves primarily originate in the proximal ureter, distributing around large vessels, the adventitia and the renal pelvis’s smooth muscle. This setup, with stretch receptors and chemosensitive properties, allows volume regulation via feedback loops [46,47]. Efferent nerves densely innervate the cortex, medulla and vascular smooth muscle of arterioles, mediating vasoconstriction, sodium and water reabsorption and renin release [46].

BP regulation relies on afferent nerves altering sympathetic outflow and efferent nerves modulating the renin–angiotensin–aldosterone system [48].

## 5. Renal Denervation

First developed in 2010 by Ardian, RDN involves percutaneous ablation of sympathetic renal nerves using radiofrequency ablation, ultrasound or neurotoxins like alcohol [25,49]. This interrupts kidney–CNS sympathetic crosstalk throughout the circadian cycle [50], potentially reducing nocturnal strokes [51,52]. Due to renal nerve distribution, ultrasound and neurolytic approaches target the main renal artery, while radiofrequency ablation can also address early branches [53].

While early randomised controlled trials (RCTs) lacked sham controls until 2014 [54,55,56,57], subsequent RCTs faced challenges including device limitations, variable medication burden, clinician expertise and variability in the understanding of renal nerve anatomy [58,59,60]. From 2017, refined trial designs culminated in the 2022 Hypertension Academic Research Consortium (HARC) report, which provided recommendations in design principles and outcome definitions for studies evaluating device-based HTN therapies [61]. Based on promising results, the current European Society of Hypertension guidelines recommend RDN for patients with eGFR > 40 mL/min/1.73 m^2^ and uncontrolled BP despite drug therapy or those with drug-related side effects or true resistant HTN (class II, level B) [62].

Meta-analyses have confirmed the BP-lowering safety of catheter-based RDN [63], including one involving over 500 patients with resistant HTN [26]. Long-term data further support its durability, with the SPYRAL HTN-ON MED trial demonstrating sustained BP reductions in patients on antihypertensive medications and a 9-year follow-up study confirming persistent efficacy and safety in resistant hypertension [64,65]. Additionally, the SPYRAL HTN-OFF MED proof-of-concept trial and its pivotal follow-up study provided robust evidence that RDN significantly reduces BP even in the absence of antihypertensive medications, reinforcing its direct physiological impact on BP regulation [66,67]. Moreover, an analysis of the SPYRAL HTN-OFF MED pivotal trial highlighted the impact of RDN on hypertensive urgencies, offering further insights into its safety profile in untreated HTN [68]. Notably, despite a subsequent re-examination of the underlying model assumptions used in the SPYRAL HTN-OFF MED pivotal trial, Böhm et al. still concluded that the effect estimate was robust to assumptions, further supporting RDN as an effective treatment for resistant HTN [69].

Moreover, the disruption of both renal efferent and afferent nerves suggests broader applications to other disease states, including arrhythmias, glucose metabolism, obstructive sleep apnea, chronic kidney disease and heart failure [27,28,29,30,31]. For example, there is growing evidence on the role of the kidneys in the development of T2DM and HTN due to an underlying central SNS overactivity [70,71]. Subgroup analyses of several RDN trials demonstrate glycaemic and other metabolic improvements in T2DM patients [63,72,73,74,75]. A pilot study by Tsioufis et al. in patients with metabolic syndrome and HTN reported a reduction in sympathetic overdrive and the restoration of normal response to oral glucose loading following RDN [76]. While the DREAMS-Study found no improvements in sympathetic activity or metabolic profile following RDN, this is likely due to insufficient denervation, the lack of a control group and a small cohort [65]. Although such studies are preliminary, HARC advocates for similar study design and attention to safety as seen in HTN trials, even if primary outcomes differ [61].

Notably, a recent study by Sharpe et al. evaluated radiofrequency RDN’s lifetime cost-effectiveness, using HTN trial data. It concluded that catheter-based RDN offers a cost-effective strategy for managing uncontrolled HTN in the UK, with a low incremental cost-effectiveness ratio, supporting its wider adoption [77].

## 6. Hepatic Innervation

**Figure 2:**
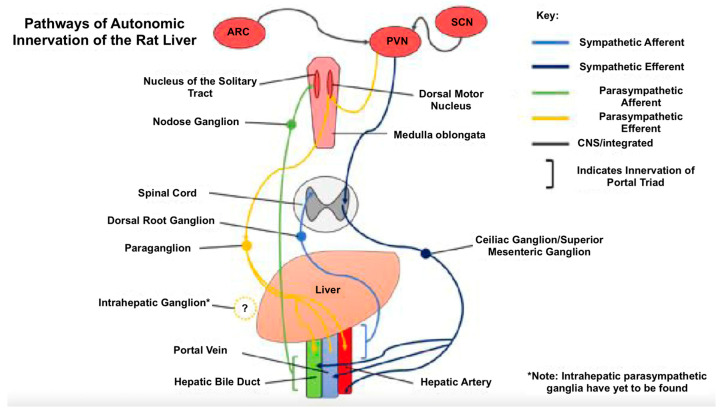
The origins and pathways of hepatic autonomic innervation in the rat liver, involving sympathetic innervation from the celiac plexus and superior mesenteric ganglia, and parasympathetic innervation from the vagus [78].

The liver is innervated by both sympathetic and parasympathetic systems. Sympathetic fibres originate from the celiac and superior mesenteric ganglia, while parasympathetic fibres branch off the vagus nerve, surrounding the portal vein, hepatic artery and bile duct (Figure 2) [78]. Afferent fibres relay information about osmolality, glucose and lipid levels in the portal vein to the CNS, while efferent fibres regulate metabolism, blood flow and bile secretion [79].

Within the portal vein, GLUT2 receptors in the liver act as glucose sensors, triggering afferent vagal fibres to signal for an increase in food intake when glucose is low. This additionally activates sympathetic fibres, promoting glycogenolysis and inhibiting glycogen synthesis, while also stimulating glucagon secretion in the pancreas [79]. Conversely, high blood glucose does not suppress food intake but activates afferent vagal fibres via hormones like glucose-like peptide, influencing insulin release and glycogenolysis [79]. Insulin’s hypoglycemic effects occur via hepatic insulin sensitising substance (HISS) and direct insulin action [79].

Lipid detection in the portal vein triggers afferent vagal fibres, decreasing food intake. The hepatorenal reflex also detects hypertonicity, reducing sodium reabsorption in the kidneys through reduced renal sympathetic tone. Additionally, hepatic nerves use ion channels like the transient receptor potential vanilloid 4 to signal hypotonicity to the brain, inducing a pressor reflex [79,80].

Efferent autonomic control regulates liver blood flow: sympathetic stimulation via adrenaline and substance P contracts the hepatic artery and reduces sinusoidal permeability, reducing blood flow, while parasympathetic activation relaxes sinusoids to aid digestion [81,82].

## 7. Anatomical Considerations: Hepatic Denervation

Tzafriri et al. examined the nerves and surrounding anatomical structures of the human common hepatic artery (CHA), with the aim of informing catheter-based denervation. Their findings indicate that the hepatic artery represents a promising and readily accessible target for sympathetic denervation procedures [34]. Moreover, an advantage to denervating the CHA is that it only supplies 10–20% of total blood flow to hepatic tissue while the portal vein supplies over 80% of all hepatic flow, suggesting that denervating the CHA would not significantly compromise overall liver perfusion [83,84].

Approximately 90% of the nerve fibres surrounding the CHA are within 5 mm of the lumen, analogous to the renal vasculature where over 80% of the nerve fibres are within 2 mm. Such anatomical proximity provides a suitable target for ablation procedures [85]. Of course, while the CHA is densely innervated by the SNS, nerve bundles also contain parasympathetic fibres, which must be considered when evaluating potential adverse effects [82].

Additionally, the CHA lumen has a 3–9 mm diameter [86]. In comparison to the renal artery, the larger lumen means that there is scope for using larger catheter sizes with lower risk of arterial spasm [85]. However, a larger lumen diameter necessitates higher heat energy levels for ablation with potential side effects including intraperitoneal bleeding, hepatic abscess, bile duct injury and gastrointestinal perforation, something which will need to be accounted for in the design of catheters [87]. Furthermore, as suggested by Kiuchi et al., some nerves may be protected from radiofrequency energy by surrounding adipose and lymph nodes, requiring depth lesions facilitated by a cooling system for effective denervation [88].

## 8. Hepatic Denervation Studies

T2DM is frequently associated with excessive activation of the SNS, which exacerbates hyperglycemia and potentiates further complications. Numerous studies in both experimental animals and humans have shown that SNS overstimulation in the liver results in heightened glucose release, leading to elevated serum glucose levels [89]. The role of the SNS in metabolic dysfunction is well documented, with evidence suggesting that increased sympathetic activation is a significant factor in the pathophysiology of both metabolic dysfunction-associated fatty liver disease (MAFLD) and non-alcoholic fatty liver disease (NAFLD) [90,91].

As shown by Kraft et al., surgical sympathetic denervation of the CHA improves glucose tolerance in high-fat/high-fructose-fed dogs, with an effect duration of at least 3 months [92]. Similarly, Wange et al. used an identical canine model to demonstrate that endovascular denervation of the CHA yielded similar results by enhancing insulin secretion and sensitivity [32]. Additionally, a recent study by Zhou et al. found that hepatic denervation restores liver regeneration under chronic stress in mice, further supporting the multifaceted benefits of hepatic denervation [33]. These findings collectively provide a strong rationale for the potential clinical application of hepatic denervation, not only for metabolic improvements but also for promoting liver regeneration.

In human participants, the COMPLEMENT study is an ongoing, prospective, first-in-human, multi-centre, non-randomised trial (NCT02278068). It aims to evaluate the initial safety and efficacy of radiofrequency ablation along the hepatic artery in patients with poorly controlled T2DM despite treatment with multiple medications. While preliminary findings support its safety profile along 46 subjects, reports of its efficacy in achieving glycaemic control are pending. Similarly, the proposed Global Pilot Study of rEnal and hepatic coMbINed denervatIon (SPYRAL GEMINI Pilot Study) aims to assess the safety and efficacy of combined hepatic and renal artery ablation in patients with uncontrolled HTN, both with and without antihypertensive medication. Further ongoing in-human multi-arm studies such as MODUS (NCT04917393), DeLIVER NZ (ACTRN12619001524189) and MILESTONE (NCT04086043) are looking into the impact of multi-organ neuromodulation on glycaemic control and BP (see ‘multiorgan neuromodulation in practice’).

Aside from its effects on glucoregulation, Bruinstroop et al. showed that hepatic sympathetic denervation enhances the lipid profile in obese Zucker rats [93]. Hurr et al. showed that liver sympathetic nerve activity doubled in obese mice and that hepatic steatosis was alleviated by sympathetic denervation and improved triglyceride accumulation pathways independent of changes in body weight, caloric intake or adiposity [94]. Therefore, hepatic denervation may have an impact on sympathetically driven liver steatosis [35].

Additionally, the ANS is also implicated in hepatic cirrhosis [95]. Liver damage typically triggers the parasympathetic activation of hepatic oval cells (HOC) to aid in the repair process [82]. Studies on rats have shown that liver regeneration after partial hepatectomy was hindered following vagotomy, highlighting the crucial role of the PNS [96]. Conversely, activation of the SNS induces changes in hepatic stellate cells, leading them to transform into myofibroblasts and contribute to fibrosis [97]. These findings align with the characteristics of cirrhotic livers, characterised by an imbalance between parasympathetic and sympathetic signalling [98]. Consequently, denervation targeting the SNS may offer potential treatment for hepatic cirrhosis, especially as suppressing this system has been found to increase HOC numbers [82].

The safety of hepatic artery denervation is supported by extensive experience with orthotropic human liver transplantation, in which there is a complete disruption of innervation. Recipients have not shown any evidence of abnormal liver function, and no significant deleterious effects on bile secretion, liver regeneration or hepatic blood flow have been documented [99].

## 9. Splenic Innervation

The spleen is innervated by the splenic nerve, which comprises autonomic nerve fibres originating from the celiac and superior mesenteric plexuses. Although fibres of the vagus nerve end in the celiac-superior mesenteric plexus, the splenic nerve has been found to contain only catecholaminergic fibres from the SNS. Therefore, it has been hypothesised that the catecholaminergic splenic nerve conveys the parasympathetic signal to the spleen [100].

Visceral afferent fibres are relayed to the brain via the celiac plexus. Sympathetic efferent fibres of the splenic plexus innervate vascular smooth muscle and tissues of the spleen, as well as the pancreas and the upper and greater curvature of the stomach, in order to control immune modulation [101,102]. Current data suggest that the association of the paraventricular nucleus and the central nucleus of the amygdala with the splenic plexus and vagus nerve may play an important modulatory role in the brain–spleen axis [103]. However, the precise details of the pathways constituting the brain–spleen axis remain poorly understood [102].

### Anatomic Considerations: Splenic Denervation

**Figure 3:**
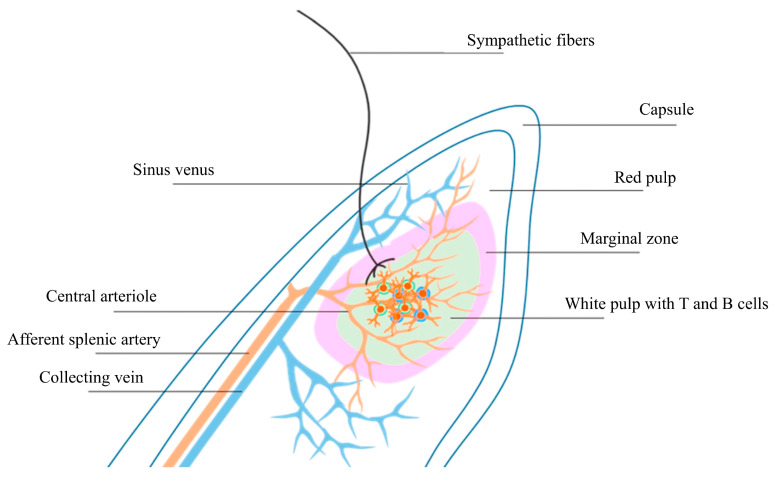
Schematic description of the architectural organisation of the spleen containing three main types of immune tissue: white pulp, red pulp and marginal zones. Sympathetic nerve fibres travel alongside the splenic artery to innervate the marginal zones [104].

Nerves from the splenic plexus travel along the splenic artery and its branches (Figure 3) [104]. Studies describe the mean diameter of the splenic artery at its origin as 5.92 mm (±1.2), with a range of 2.81 mm to 10.84 mm, while the middle splenic artery diameter is reported to be 5.92 mm (±1.2), with a range of 2.81 mm to 10.84 mm [105]. Approximately 50% of nerve fibres are found within 0–1 mm of the lumen, 30% within 1–2 mm and 15% within 2–3 mm, with the remaining 5% found in the 3–4 mm circumferential region around the artery [106].

Cleypool et al. reported that the splenic artery was straight in 15% of human participants, slightly curved in 45% and looped or coiled in 40%. In fact, one or multiple loops were observed in 83% of the studied specimens, which could prove challenging when navigating a catheter for ablation purposes [107]. Therefore, the variability in diameter and course highlights the importance of considering individual anatomical differences when performing procedures or interpreting radiographic studies involving this vessel.

## 10. Splenic Denervation Studies

Splenic artery denervation, still in pre-clinical stages, shows potential for neuromodulation due to the link between splenic SNS overactivity, PNS underactivity and various diseases [108]. Studies in small animal models of HTN show regional sympathetic activation, more pronounced in the splanchnic than renal nervous system [36,109]. Furthermore, catheter-based splanchnic denervation reduced venoarterial norepinephrine gradients over the splanchnic organs associated with sympathetic splenic innervation. This subsequently alleviated HTN, left ventricular remodelling and hypertrophy and preserved left ventricular function in pigs with hypertrophic cardiomyopathy [37].

Rheumatoid arthritis is associated with increased sympathetic activity and altered hypothalamic–pituitary–adrenal axis function [110]. Donega et al. demonstrated that spleen-targeted autonomic nerve stimulation can modulate immune responses in pigs [111]. Albaghdadi et al. revealed that splenic denervation reduced norepinephrine in both naïve and inflammatory arthritis pigs, but did not significantly affect joint injury or inflammatory markers, indicating the need for further research [112].

Interestingly, patients with post-traumatic stress disorder (PTSD) often exhibit heightened sympathetic tone, and increased inflammatory risks are common [113]. A murine model of repeated social defeat stress (RSDS) that replicates the behavioural, autonomic and inflammatory aspects of PTSD demonstrated that while splenic denervation did not alter behaviour, it reduced inflammation, T-lymphocyte mitochondrial superoxide, T-helper 17 polarisation and pro-inflammatory gene expression [114]. These findings suggest that SNS modulation may affect neuroimmune interactions. However, as with Albaghdadi’s study on arthritic pigs, the presence of biochemical findings and the lack of clinical impact suggest that further research is warranted.

## 11. Other Denervation Sites

### 11.1. Pulmonary Artery Denervation

**Figure 4:**
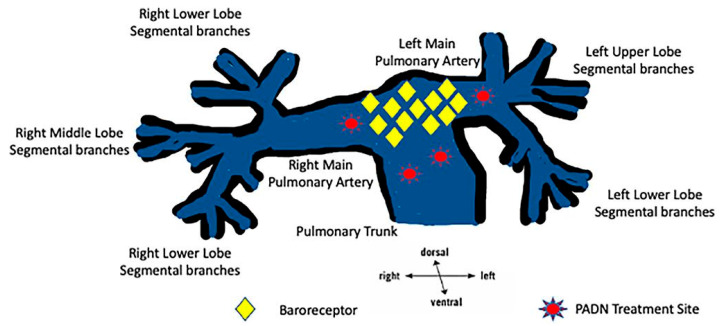
A graphic of the pulmonary arterial tree showing the common locations of baroreceptors and the current pulmonary artery denervation treatment sites in the pulmonary trunk, the pulmonary bifurcation and the pulmonary arteries [116].

Pulmonary arterial hypertension (PAH) is a rare, progressive disorder characterised by HTN in the arteries of the lungs (pulmonary artery). It encompasses a range of clinical entities and can be idiopathic, drug-induced, heritable or associated with underlying systemic disease. The SNS appears to play a major role in the increased vascular tone that is present in PAH [20]. In fact, the adventitia of pulmonary vessels receive a rich autonomic nerve supply, with predominantly sympathetic but also parasympathetic and sensory nerve fibres (Figure 4) [115,116].

In an animal study by Rothman et al., pulmonary artery denervation (PADN) led to an immediate drop in mean pulmonary artery pressure [117]. Subsequently, Chen et al. conducted the first PADN clinical study in low-risk idiopathic PAH patients, finding that pulmonary artery pressure declined and that cardiac output and 6 min walk distance (6MWD) improved at three months [118]. Later studies confirmed the efficacy of PADN on the internal surface of the pulmonary artery in patients with different causes of PAH at different centres [119,120].

A recent study (TROPHY1) validated the safety and efficacy of using high-frequency ultrasound for PADN in PAH patients [38]. In the latest TROPHY1 study, which used an intravascular ultrasound catheter, PADN resulted in significant reductions in pulmonary vascular resistance, as well as increased 6MWD and daily activity without any adverse events [121]. Additionally, the latest PADN-5 study on patients with both pre- and post-capillary PAH showed that PADN led to an increase in 6MWD and a decrease in pulmonary vascular resistance and pulmonary artery wedge pressure compared to sham denervation plus sildenafil therapy groups [120]. Similarly, a study by Romanov et al. compared PADN with medical therapy for residual PAH after pulmonary endarterectomy in patients with chronic thromboembolic pulmonary hypertension. At 12 months, the PADN group showed a significant reduction in pulmonary vascular resistance and an increase in 6MWD, while the medical therapy group had more frequent clinical worsening [122].

Existing research has yielded encouraging results regarding the safety and effectiveness of PADN procedures [116]. However, further investigation that considers the careful selection of homogenous patient cohorts to delineate potential confounding factors is warranted.

### 11.2. Carotid Body Denervation

**Figure 5:**
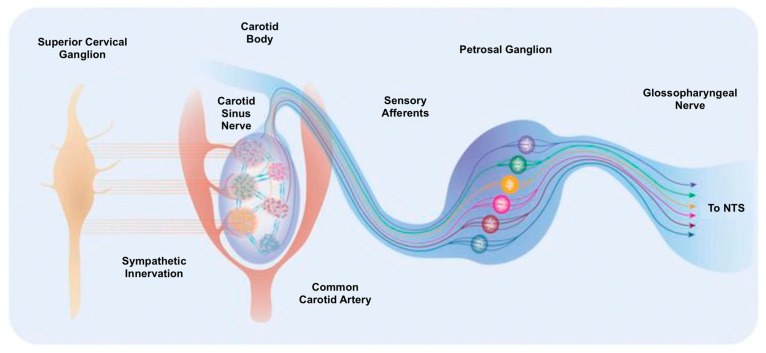
Carotid body location, innervation and connectivity. The carotid bodies reside in the bifurcation of the common carotid artery bilaterally and are highly vascularised [123].

The carotid body, a small yet highly vascularized organ, plays a crucial role in regulating physiological responses to hypoxia. Located near the carotid sinus at the bifurcation of the common carotid artery (Figure 5) [123,124], this organ is about 4 mm in diameter in humans. It is innervated by sensory fibres originating from the petrosal ganglion. The carotid body is composed mainly of two cell types: glomus cells, which detect oxygen levels and respond to hypoxia, and sustentacular cells, which encase the glomus cells in a glia-like manner [125]. Though traditionally viewed as supportive, recent research suggests that sustentacular cells may also contribute to sensory signalling and could act as stem cells replenishing the glomus cell population [126,127,128].

This organ’s role extends beyond its oxygen-sensing function. It is central to the body’s response to hypoxia, triggering increases in sympathetic nerve activity, BP, respiration and gluconeogenesis. In pathological conditions like obesity, chronic intermittent hypoxia leads to overactivity of the carotid body, contributing to metabolic dysfunction. This overactivation is a potential target for therapies aimed at mitigating the burden of cardiometabolic diseases [124].

Research has shown that overactivation of the carotid body is linked to insulin resistance [39]. In animal models of obesity, for instance, Shin et al. studied obese male mice subjected to chronic intermittent hypoxia. Mice that underwent carotid sinus nerve dissection exhibited improved insulin sensitivity, suggesting that carotid body activity directly influences glucose regulation. Similarly, Sacramento et al. demonstrated that severing the carotid sinus nerves in rats resulted in enhanced insulin sensitivity and reduced fasting blood glucose levels. Further experiments with bioelectric modulation in rats fed a high fat and fructose diet indicated that insulin sensitivity improved with nerve denervation but deteriorated once nerve activity was restored to its heightened state [129].

Beyond its metabolic effects, the carotid body is increasingly implicated in sympathoexcitatory conditions such as congestive heart failure (CHF) and sleep apnoea [40]. Marcus et al. investigated the impact of carotid body denervation in rabbits with CHF induced by ventricular pacing. Nine days following the procedure, the rabbits showed significant recovery in several cardiac and autonomic parameters, which are usually impaired in CHF [130].

Although research on carotid body denervation is still in its early stages, these findings highlight its potential as a therapeutic strategy. However, more studies are required to assess its safety and efficacy before it can be considered for clinical application in humans.

## 12. Multiorgan Neuromodulation in Practice

Ongoing research into the pathophysiological mechanisms underlying sympathetic denervation may eventually lead to the precise identification of the optimal combination of arteries for denervation, enabling effective treatment for a wide range of patients with diverse medical conditions. Well-designed trials should perform denervation procedures for each organ separately and in combination without confounding medication for a limited period under rigorous medical care and oversight, while also including a sham control arm. Currently, there are over 100 registered randomised controlled trials focused on investigating novel denervation approaches [131]. However, there is a scarcity of trials examining multi-organ denervation.

Kiuchi et al. investigated the feasibility, safety and efficacy of endovascular denervation of both hepatic and renal arteries in a swine model, achieving significant and sustained denervation without major safety events over 30 and 90 days of follow-up. Norepinephrine concentrations were significantly reduced in the kidneys (−78%, *p*  <  0.001), liver (−88%, *p*  =  0.005), pancreas (−78%, *p*  =  0.018) and duodenum (−95%, *p*  =  0.028) following multi-organ denervation treatment compared to control animals, with favourable tissue responses confirmed through histological findings at 90 days follow-up [74].

Given that the prevalence of sympathetic overactivity is estimated to be as high as 80% among those with cardiometabolic syndrome, multi-organ denervation may play a role in ‘interventional metabology’ through alleviating issues pertaining to additional parameters including hyperglycemia, hypercholesterolaemia and even sleep apnoea [132]. There are four major upcoming multi-organ denervation trials in humans: DeLIVER NZ (ACTRN12619001524189), MODUS (NCT04917393), SPYRAL GEMINI (upcoming) and MILESTONE (NCT04086043).

The DeLIVER NZ study from New Zealand evaluates the safety of renal and CHA denervation using the Metavention iRF Denervation System for HTN. Additionally, the MODUS feasibility study assesses hepatic, renal or multi-organ (hepatic and renal) denervation with the same system for T2DM and HTN, with preliminary results showing a 20 mmHg BP reduction—greater than renal artery ablation alone. Similarly, the recently announced SPYRAL GEMINI study will investigate multi-organ denervation in uncontrolled HTN using the Symplicity Spyral catheter, with and without antihypertensive therapy. Another study, MILESTONE is evaluating the safety and glycaemic effects of multi-electrode endovascular denervation of the coeliac artery in T2DM, with early findings suggesting significant improvement in hyperglycaemia and good tolerability.

## 13. Potential Side Effects

Despite the perceived therapeutic benefits of multimodal sympathetic denervation, there are concerns stemming from potential unknown side effects. Due to the comprehensive innervation of celiac sympathetic nerves, the effects on different organs need to be clearly understood to optimise ablation sites and reduce complications [133]. Moreover, targeted denervation results in the ablation of nerve bundles that may contain a mixture of sympathetic and parasympathetic fibres such as the CHA. While parasympathetic denervation presumably results in the opposite effect to its physiological function, the true clinical consequences of parasympathetic denervation remain unknown [134,135].

For example, Brinkman et al. illustrated that splenic nerve bundle stimulation, which involves norepinephrine release, resulted in reduced clinical features of colonic inflammation in mice with dextran sodium sulphate-induced colitis [136]. They hypothesised that this action may have resulted from immunosuppression due to inhibited splenic myeloid cell activation. Although this was a murine model, the opposing impact of splenic denervation among individuals with inflammatory bowel disease is now questionable and may require extra caution in this group of patients.

Additionally, orthotopic liver transplant recipients display increased obesity, dyslipidemia, HTN and diabetes mellitus rates following liver transplantation, suggesting metabolic dysfunctions that may be caused by denervation of the liver [137]. While other confounding factors may be contributing to these confusing observations, careful long-term follow-up of patients undergoing hepatic denervation is vital.

## 14. Important Considerations

Before the application of multimodal sympathetic denervation, it is important to address several issues. First, we must identify patients who would respond effectively to denervation. Patients with higher baseline sympathetic activity may be ideal candidates; however, this is difficult to establish [138]. Perhaps responsive patients could include those who are truly ‘resistant’ to anti-hypertensives or those who develop HTN from an early age. A study by Kantauskaite et al. demonstrated that patients with resistant HTN had a higher burden of T-cell signatures at baseline than controls and that those with the highest burden demonstrated a larger BP reduction following RDN [139]. This may provide a useful method of selecting patients for renal or other organ denervation. Importantly, we also do not know whether patients who already have end-organ damage will be as responsive to sympathetic denervation [140,141].

Secondly, while research has been conducted to study optimal ablation parameters in RDN, extensive data are required for radiofrequency ablation in other arteries. After considering general anatomical variations, it is essential to precisely locate nerve bundles to ensure both effectiveness and safety. Moreover, the dose–response relationship requires clarification, especially as we introduce multimodal neuromodulation and because previous RCT parameters have limited the maximum drop in BP [142,143,144,145].

Thirdly, due to sample size limitations, RDN has relied on indirect cardiovascular outcome data that relies on BP as a surrogate outcome through the interpolation of trial endpoints with those of pharmacological trials [53]. For example, in a large-scale meta-analysis of pharmacological therapy for HTN, a 10 mmHg drop in systolic BP was associated with a significantly reduced major adverse cardiac event risk (RR 0.80, 95% CI 0.77–0.83) [146]. Future extra-renal or multimodal denervation trials that focus on other outcomes such as glycaemic control may require interpolation with outcome data from other trials as patient numbers will undoubtedly be insufficiently powered due to small sample sizes.

## 15. Conclusions

In conclusion, although still in its early stages, multi-organ denervation offers promise for treating a wide array of diseases, including cardio-metabolic abnormalities and inflammatory conditions. Given that the efficacy, long-term safety and cost-effectiveness of RDN have led to its implementation as a complementary intervention for HTN in various guidelines, the targeted sympathetic ablation of additional organs may provide more effective BP control. Such an intervention may also help overcome challenges related to medication adherence among patients.

However, organ denervation has limitations, including its invasive nature and associated risks such as vascular complications and potential effects on surrounding neural structures. Additionally, uncertainties remain regarding its long-term efficacy and durability. In contrast, advancements in pharmacologic therapies provide a non-invasive alternative with improved efficacy and safety profiles, allowing flexible adjustments in treatment based on patient response.

Of course, further research is needed to identify individuals who would respond best to denervation, optimise procedural factors and assess cost-effectiveness. Therefore, additional preclinical experiments and carefully designed clinical trials are still required to fully understand the potential risks and benefits of multi-organ denervation. Ultimately, this approach should be considered within a comprehensive treatment framework, weighing its benefits and risks against those of newer pharmacologic options.

## Figures and Tables

**Table 1:** Examples of target denervation sites and reported/potential clinical impact.

Denervation Site(s)	Targeted Condition
Renal artery	Hypertension [26]
	Arrhythmia [27]
	Dysglycaemia [28]
	Obstructive sleep apnoea [29]
	Chronic kidney disease [30]
	Heart failure [31]
Common hepatic artery	Dysglycaemia [32]
	Dyslipidaemia/insulin resistance [33]
	Hypertension [34]
	Liver cirrhosis [35]
Splenic artery	Hypertension [36]
	Hypertrophic cardiomyopathy [37]
	Rheumatoid arthritis [37]
Pulmonary artery	Pulmonary arterial hypertension [38]
Carotid body	Dysglycaemia/insulin resistance [39]
	Heart failure [40]
	Obstructive sleep apnoea [40]

## Data Availability

Not applicable.

## References

[1] McCorry L.K. (2007). Physiology of the Autonomic Nervous System. Am. J. Pharm. Educ..

[2] Chu B., Marwaha K., Sanvictores T., Awosika A.O., Ayers D. (2024). Physiology, Stress Reaction. StatPearls.

[3] Carnagarin R., Lambert G.W., Kiuchi M.G., Nolde J.M., Matthews V.B., Eikelis N., Lambert E.A., Schlaich M.P. (2019). Effects of sympathetic modulation in metabolic disease. Ann. N. Y. Acad. Sci..

[4] Hillebrand S., De Mutsert R., Christen T., Maan A.C., Jukema J.W., Lamb H.J., De Roos A., Rosendaal F.R., Den Heijer M., Swenne C.A. (2014). Body fat, especially visceral fat, is associated with electrocardiographic measures of sympathetic activation: Visceral Fat and Sympathetic Activation. Obesity.

[5] Rahman A., Hasan A., Nishiyama A., Kobori H. (2018). Altered Circadian Timing System-Mediated Non-Dipping Pattern of Blood Pressure and Associated Cardiovascular Disorders in Metabolic and Kidney Diseases. Int. J. Mol. Sci..

[6] Narkiewicz K., Van De Borne P.J.H., Montano N., Dyken M.E., Phillips B.G., Somers V.K. (1998). Contribution of Tonic Chemoreflex Activation to Sympathetic Activity and Blood Pressure in Patients with Obstructive Sleep Apnea. Circulation.

[7] Somers V.K., Dyken M.E., Clary M.P., Abboud F.M. (1995). Sympathetic neural mechanisms in obstructive sleep apnea. J. Clin. Investig..

[8] Mansukhani M.P., Kara T., Caples S.M., Somers V.K. (2014). Chemoreflexes, Sleep Apnea, and Sympathetic Dysregulation. Curr. Hypertens. Rep..

[9] Borovac J.A., D’Amario D., Bozic J., Glavas D. (2020). Sympathetic nervous system activation and heart failure: Current state of evidence and the pathophysiology in the light of novel biomarkers. World J. Cardiol..

[10] Zhang D.Y., Anderson A.S. (2014). The Sympathetic Nervous System and Heart Failure. Cardiol. Clin..

[11] Velez-Roa S., Ciarka A., Najem B., Vachiery J.-L., Naeije R., Van De Borne P. (2004). Increased Sympathetic Nerve Activity in Pulmonary Artery Hypertension. Circulation.

[12] Chaudhary K., Buddineni J.P., Nistala R., Whaley-Connell A. (2011). Resistant Hypertension in the High-Risk Metabolic Patient. Curr. Diabetes Rep..

[13] McLaughlin T., Abbasi F., Cheal K., Chu J., Lamendola C., Reaven G. (2003). Use of Metabolic Markers To Identify Overweight Individuals Who Are Insulin Resistant. Ann. Intern. Med..

[14] Kiuchi M.G., Carnagarin R., Matthews V.B., Schlaich M.P. (2023). Multi-organ denervation: A novel approach to combat cardiometabolic disease. Hypertens. Res..

[15] Bellocchi C., Carandina A., Montinaro B., Targetti E., Furlan L., Rodrigues G.D., Tobaldini E., Montano N. (2022). The Interplay between Autonomic Nervous System and Inflammation across Systemic Autoimmune Diseases. Int. J. Mol. Sci..

[16] Chakravarthy K., Chaudhry H., Williams K., Christo P.J. (2015). Review of the Uses of Vagal Nerve Stimulation in Chronic Pain Management. Curr. Pain Headache Rep..

[17] Napadow V., Edwards R.R., Cahalan C.M., Mensing G., Greenbaum S., Valovska A., Li A., Kim J., Maeda Y., Park K. (2012). Evoked Pain Analgesia in Chronic Pelvic Pain Patients Using Respiratory-Gated Auricular Vagal Afferent Nerve Stimulation. Pain Med..

[18] Janner H., Klausenitz C., Gürtler N., Hahnenkamp K., Usichenko T.I. (2018). Effects of Electrical Transcutaneous Vagus Nerve Stimulation on the Perceived Intensity of Repetitive Painful Heat Stimuli: A Blinded Placebo- and Sham-Controlled Randomized Crossover Investigation. Anesth. Analg..

[19] Vrijens B., Antoniou S., Burnier M., De La Sierra A., Volpe M. (2017). Current Situation of Medication Adherence in Hypertension. Front. Pharmacol..

[20] Burnier M., Egan B.M. (2019). Adherence in Hypertension: A Review of Prevalence, Risk Factors, Impact, and Management. Circ. Res..

[21] Blaschke T.F., Osterberg L., Vrijens B., Urquhart J. (2012). Adherence to Medications: Insights Arising from Studies on the Unreliable Link Between Prescribed and Actual Drug Dosing Histories. Annu. Rev. Pharmacol. Toxicol..

[22] Noubiap J.J., Nansseu J.R., Lontchi-Yimagou E., Nkeck J.R., Nyaga U.F., Ngouo A.T., Tounouga D.N., Tianyi F.-L., Foka A.J., Ndoadoumgue A.L. (2022). Geographic distribution of metabolic syndrome and its components in the general adult population: A meta-analysis of global data from 28 million individuals. Diabetes Res. Clin. Pract..

[23] Kandzari D.E. (2021). Catheter-Based Renal Denervation Therapy: Evolution of Evidence and Future Directions. Circ. Cardiovasc. Interv..

[24] Smithwick R.H. (1953). Splanchnicectomy for Essential Hypertension: Results in 1266 Cases. J. Am. Med. Assoc..

[25] Symplicity HTN-2 Investigators (2010). Renal sympathetic denervation in patients with treatment-resistant hypertension (The Symplicity HTN-2 Trial): A randomised controlled trial. Lancet.

[26] Davis M.I., Filion K.B., Zhang D., Eisenberg M.J., Afilalo J., Schiffrin E.L., Joyal D. (2013). Effectiveness of renal denervation therapy for resistant hypertension: A systematic review and meta-analysis. J. Am. Coll. Cardiol..

[27] Zhang W., Zhou Q., Lu Y., Li Y., Zhang L., Zhang J., Xing Q., Lv W., Cheng X., Zhang G. (2018). Renal Denervation Reduced Ventricular Arrhythmia After Myocardial Infarction by Inhibiting Sympathetic Activity and Remodeling. J. Am. Heart Assoc..

[28] Verloop W.L., Spiering W., Vink E.E., Beeftink M.M.A., Blankestijn P.J., Doevendans P.A., Voskuil M. (2015). Denervation of the Renal Arteries in Metabolic Syndrome: The DREAMS-Study. Hypertension.

[29] Warchol-Celinska E., Prejbisz A., Kadziela J., Florczak E., Januszewicz M., Michalowska I., Dobrowolski P., Kabat M., Sliwinski P., Klisiewicz A. (2018). Renal Denervation in Resistant Hypertension and Obstructive Sleep Apnea: Randomized Proof-of-Concept Phase II Trial. Hypertension.

[30] Schmieder R.E. (2023). Renal denervation in patients with chronic kidney disease: Current evidence and future perspectives. Nephrol. Dial. Transplant..

[31] Fukuta H., Goto T., Wakami K., Ohte N. (2017). Effects of catheter-based renal denervation on heart failure with reduced ejection fraction: A systematic review and meta-analysis. Heart Fail. Rev..

[32] Wang Z., Zhu D., Zhu X., Liu D., Cao Q., Pan T., Zhang Q., Gu X., Li L., Teng G. (2023). Interventional metabology: A review of bariatric arterial embolization and endovascular denervation for treating metabolic disorders. J. Diabetes.

[33] Zhou Y., Lin X., Jiao Y., Yang D., Li Z., Zhu L., Li Y., Yin S., Li Q., Xu S. (2024). A brain-to-liver signal mediates the inhibition of liver regeneration under chronic stress in mice. Nat. Commun..

[34] Tzafriri A.R., Garcia-Polite F., Keating J., Melidone R., Knutson J., Markham P., Edelman E.R., Mahfoud F. (2022). Morphometric analysis of the human common hepatic artery reveals a rich and accessible target for sympathetic liver denervation. Sci. Rep..

[35] Ferris H.A., Kahn C.R. (2016). Unraveling the Paradox of Selective Insulin Resistance in the Liver: The Brain-Liver Connection. Diabetes.

[36] Kandlikar S.S., Fink G.D. (2011). Splanchnic sympathetic nerves in the development of mild DOCA-salt hypertension. Am. J. Physiol. Heart Circ. Physiol..

[37] Zhen Z., Liao S.-Y., Zhu Z.-Y., Sijia S., Au K.-W., Lai W.-H., Tsang A., Hai J.S.H., Tse H.-F. (2019). Catheter-Based Splanchnic Denervation for Treatment of Hypertensive Cardiomyopathy. Hypertension.

[38] Rothman A.M.K., Vachiery J.-L., Howard L.S., Mikhail G.W., Lang I.M., Jonas M., Kiely D.G., Shav D., Shabtay O., Avriel A. (2020). Intravascular Ultrasound Pulmonary Artery Denervation to Treat Pulmonary Arterial Hypertension (TROPHY1). JACC Cardiovasc. Interv..

[39] Ribeiro M.J., Sacramento J.F., Gonzalez C., Guarino M.P., Monteiro E.C., Conde S.V. (2013). Carotid body denervation prevents the development of insulin resistance and hypertension induced by hypercaloric diets. Diabetes.

[40] Johnson B.D., Limberg J.K. (2014). Is carotid body denervation the silver bullet for heart failure?. J. Physiol..

[41] Waxenbaum J.A., Reddy V., Varacallo M. (2024). Anatomy, Autonomic Nervous System. StatPearls.

[42] Sanvictores T., Jozsa F., Tadi P. (2024). Neuroanatomy, Autonomic Nervous System Visceral Afferent Fibers and Pain. StatPearls.

[43] McCausland C., Carey F.J., Sajjad H. (2024). Anatomy, Back, Splanchnic Nerve. StatPearls.

[44] García-Touchard A., Sañudo J.R. (2019). Renal Denervation. Importance of Knowledge of Sympathetic Nervous System Anatomy in Refining the Technique. Rev. Esp. Cardiol. Engl. Ed..

[45] Leslie S.W., Sajjad H. (2024). Anatomy, Abdomen and Pelvis, Renal Artery. StatPearls.

[46] Sata Y., Head G.A., Denton K., May C.N., Schlaich M.P. (2018). Role of the Sympathetic Nervous System and Its Modulation in Renal Hypertension. Front. Med..

[47] Hering L., Rahman M., Potthoff S.A., Rump L.C., Stegbauer J. (2020). Role of α2-Adrenoceptors in Hypertension: Focus on Renal Sympathetic Neurotransmitter Release, Inflammation, and Sodium Homeostasis. Front. Physiol..

[48] Fountain J.H., Kaur J., Lappin S.L. (2024). Physiology, Renin Angiotensin System. StatPearls.

[49] Rey-García J., Townsend R.R. (2022). Renal Denervation: A Review. Am. J. Kidney Dis..

[50] Katsurada K., Shinohara K., Aoki J., Nanto S., Kario K. (2022). Renal denervation: Basic and clinical evidence. Hypertens. Res..

[51] Castagna F., Mondellini G.M., Pinsino A., McDonnell B.J., Stöhr E.J., Gaudig A., Amlani A., Nwokocha J., Te-Frey R., Takeda K. (2020). Lack of Nocturnal Blood Pressure Reduction Increases the Risk of Stroke in Patients on Left Ventricular Assist Device Support. J. Heart Lung Transplant..

[52] Nadeau J.O., Fang J., Kapral M.K., Silver F.L., Hill M.D. (2005). Outcome after Stroke upon Awakening. Can. J. Neurol. Sci..

[53] Haider S.A., Wagener M., Iqbal T., Shahzad S., Del Sole P.A., Leahy N., Murphy D., Sharif R., Ullah I., Sharif F. (2024). Does renal denervation require cardiovascular outcome-driven data?. Hypertens. Res..

[54] Esler M.D., Krum H., Schlaich M., Schmieder R.E., Böhm M., Sobotka P.A. (2012). Renal Sympathetic Denervation for Treatment of Drug-Resistant Hypertension: One-Year Results from the Symplicity HTN-2 Randomized, Controlled Trial. Circulation.

[55] Azizi M., Sapoval M., Gosse P., Monge M., Bobrie G., Delsart P., Midulla M., Mounier-Véhier C., Courand P.-Y., Lantelme P. (2015). Optimum and stepped care standardised antihypertensive treatment with or without renal denervation for resistant hypertension (DENERHTN): A multicentre, open-label, randomised controlled trial. Lancet.

[56] Fengler K., Rommel K.-P., Blazek S., Besler C., Hartung P., Von Roeder M., Petzold M., Winkler S., Höllriegel R., Desch S. (2019). A Three-Arm Randomized Trial of Different Renal Denervation Devices and Techniques in Patients with Resistant Hypertension (RADIOSOUND-HTN). Circulation.

[57] Bhatt D.L., Kandzari D.E., O’Neill W.W., D’Agostino R., Flack J.M., Katzen B.T., Leon M.B., Liu M., Mauri L., Negoita M. (2014). A Controlled Trial of Renal Denervation for Resistant Hypertension. N. Engl. J. Med..

[58] Kandzari D.E., Bhatt D.L., Brar S., Devireddy C.M., Esler M., Fahy M., Flack J.M., Katzen B.T., Lea J., Lee D.P. (2015). Predictors of blood pressure response in the SYMPLICITY HTN-3 trial. Eur. Heart J..

[59] Sakakura K., Ladich E., Cheng Q., Otsuka F., Yahagi K., Fowler D.R., Kolodgie F.D., Virmani R., Joner M. (2014). Anatomic Assessment of Sympathetic Peri-Arterial Renal Nerves in Man. J. Am. Coll. Cardiol..

[60] Kasprzycki K., Petkow-Dimitrow P., Krawczyk-Ożóg A., Bartuś S., Rajtar-Salwa R. (2023). Anatomic Variations of Renal Arteries as an Important Factor in the Effectiveness of Renal Denervation in Resistant Hypertension. J. Cardiovasc. Dev. Dis..

[61] Kandzari D.E., Mahfoud F., Weber M.A., Townsend R., Parati G., Fisher N.D.L., Lobo M.D., Bloch M., Böhm M., Sharp A.S.P. (2022). Clinical Trial Design Principles and Outcomes Definitions for Device-Based Therapies for Hypertension: A Consensus Document from the Hypertension Academic Research Consortium. Circulation.

[62] Barbato E., Azizi M., Schmieder R.E., Lauder L., Böhm M., Brouwers S., Bruno R.M., Dudek D., Kahan T., Kandzari D.E. (2023). Renal denervation in the management of hypertension in adults. A clinical consensus statement of the ESC Council on Hypertension and the European Association of Percutaneous Cardiovascular Interventions (EAPCI). EuroIntervention.

[63] Ogoyama Y., Tada K., Abe M., Nanto S., Shibata H., Mukoyama M., Kai H., Arima H., Kario K. (2022). Effects of renal denervation on blood pressures in patients with hypertension: A systematic review and meta-analysis of randomized sham-controlled trials. Hypertens. Res..

[64] Mahfoud F., Kandzari D.E., Kario K., Townsend R.R., Weber M.A., Schmieder R.E., Tsioufis K., Pocock S., Dimitriadis K., Choi J.W. (2022). Long-term efficacy and safety of renal denervation in the presence of antihypertensive drugs (SPYRAL HTN-ON MED): A randomised, sham-controlled trial. Lancet.

[65] Sesa-Ashton G., Nolde J.M., Muente I., Carnagarin R., Lee R., Macefield V.G., Dawood T., Sata Y., Lambert E.A., Lambert G.W. (2023). Catheter-Based Renal Denervation: 9-Year Follow-Up Data on Safety and Blood Pressure Reduction in Patients with Resistant Hypertension. Hypertension.

[66] Townsend R.R., Mahfoud F., Kandzari D.E., Kario K., Pocock S., Weber M.A., Ewen S., Tsioufis K., Tousoulis D., Sharp A.S.P. (2017). Catheter-based renal denervation in patients with uncontrolled hypertension in the absence of antihypertensive medications (SPYRAL HTN-OFF MED): A randomised, sham-controlled, proof-of-concept trial. Lancet.

[67] Böhm M., Kario K., Kandzari D.E., Mahfoud F., Weber M.A., Schmieder R.E., Tsioufis K., Pocock S., Konstantinidis D., Choi J.W. (2020). Efficacy of catheter-based renal denervation in the absence of antihypertensive medications (SPYRAL HTN-OFF MED Pivotal): A multicentre, randomised, sham-controlled trial. Lancet.

[68] Weber M.A., Schmieder R.E., Kandzari D.E., Townsend R.R., Mahfoud F., Tsioufis K., Kario K., Pocock S., Tatakis F., Ewen S. (2022). Hypertension urgencies in the SPYRAL HTN-OFF MED Pivotal trial. Clin. Res. Cardiol..

[69] Böhm M., Fahy M., Hickey G.L., Pocock S., Brar S., DeBruin V., Weber M.A., Mahfoud F., Kandzari D.E. (2021). A re-examination of the SPYRAL HTN-OFF MED Pivotal trial with respect to the underlying model assumptions. Contemp. Clin. Trials Commun..

[70] Matthews V.B., Elliot R.H., Rudnicka C., Hricova J., Herat L., Schlaich M.P. (2017). Role of the sympathetic nervous system in regulation of the sodium glucose cotransporter 2. J. Hypertens..

[71] Cherrington A.D. (1999). Banting Lecture 1997. Control of glucose uptake and release by the liver in vivo. Diabetes.

[72] Mahfoud F., Schlaich M., Kindermann I., Ukena C., Cremers B., Brandt M.C., Hoppe U.C., Vonend O., Rump L.C., Sobotka P.A. (2011). Effect of Renal Sympathetic Denervation on Glucose Metabolism in Patients with Resistant Hypertension: A Pilot Study. Circulation.

[73] Kądziela J., Warchoł-Celińska E., Prejbisz A., Januszewicz A., Witkowski A., Tsioufis K. (2018). Renal denervation—Can we press the “ON” button again?. Adv. Interv. Cardiol..

[74] Pan T., Guo J., Teng G. (2015). Renal Denervation: A Potential Novel Treatment for Type 2 Diabetes Mellitus?. Medicine.

[75] Witkowski A., Prejbisz A., Florczak E., Kądziela J., Śliwiński P., Bieleń P., Michałowska I., Kabat M., Warchoł E., Januszewicz M. (2011). Effects of renal sympathetic denervation on blood pressure, sleep apnea course, and glycemic control in patients with resistant hypertension and sleep apnea. Hypertension.

[76] Tsioufis C., Dimitriadis K., Kasiakogias A., Kalos T., Liatakis I., Koutra E., Nikolopoulou L., Kordalis A., Ella R.O., Lau E.O.-Y. (2017). Effects of multielectrode renal denervation on elevated sympathetic nerve activity and insulin resistance in metabolic syndrome. J. Hypertens..

[77] Sharp A.S.P., Kinnaird T., Curzen N., Ayyub R., Alfonso J.E., Mamas M.A., Vanden Bavière H. (2024). Cost-effectiveness of intravascular ultrasound-guided percutaneous intervention in patients with acute coronary syndromes: A UK perspective. Eur. Heart J.-Qual. Care Clin. Outcomes.

[78] Negrete K. (2020). Anatomy and Function of Autonomic Innervation of the Liver. Pegasus Rev. UCF Undergrad. Res. J..

[79] Mizuno K., Ueno Y. (2017). Autonomic Nervous System and the Liver. Hepatol. Res..

[80] Yu S., Huang S., Ding Y., Wang W., Wang A., Lu Y. (2019). Transient receptor potential ion-channel subfamily V member 4: A potential target for cancer treatment. Cell Death Dis..

[81] Ueno T., Bioulac-Sage P., Balabaud C., Rosenbaum J. (2004). Innervation of the sinusoidal wall: Regulation of the sinusoidal diameter. Anat. Rec. Part A Discov. Mol. Cell. Evol. Biol..

[82] Jensen K.J., Alpini G., Glaser S. (2013). Hepatic Nervous System and Neurobiology of the Liver. Compr. Physiol..

[83] Kan Z., Madoff D. (2008). Liver Anatomy: Microcirculation of the Liver. Semin. Interv. Radiol..

[84] Eipel C., Abshagen K., Vollmar B. (2010). Regulation of hepatic blood flow: The hepatic arterial buffer response revisited. World J. Gastroenterol..

[85] Choe W.-S., Song W.H., Jeong C.W., Choi E.-K., Oh S. (2019). Anatomic Conformation of Renal Sympathetic Nerve Fibers in Living Human Tissues. Sci. Rep..

[86] Singh B.G.P., Bhatt C.R., Patel S.V., Mehta C.D. (2014). Morphometric Study of Coeliac Trunk Specific Reference to Hepatic Artery Pattern in the West-Indian Population. Indian. J. Surg..

[87] Huang Q., Pang M., Zeng Q., He X., Zheng R., Ge M., Li K. (2022). The frequency and risk factors of major complications after thermal ablation of liver tumours in 2,084 ablation sessions. Front. Surg..

[88] Kiuchi M.G., Ganesan K., Keating J., Carnagarin R., Matthews V.B., Herat L.Y., Goh G., Adams L., Schlaich M.P. (2021). Combined renal and common hepatic artery denervation as a novel approach to reduce cardiometabolic risk: Technical approach, feasibility and safety in a pre-clinical model. Clin. Res. Cardiol. Off. J. Ger. Card. Soc..

[89] Hazlehurst J.M., Woods C., Marjot T., Cobbold J.F., Tomlinson J.W. (2016). Non-alcoholic fatty liver disease and diabetes. Metabolism.

[90] Carnagarin R., Tan K., Adams L., Matthews V.B., Kiuchi M.G., Gavidia L.M.L., Lambert G.W., Lambert E.A., Herat L.Y., Schlaich M.P. (2021). Metabolic Dysfunction-Associated Fatty Liver Disease (MAFLD)—A Condition Associated with Heightened Sympathetic Activation. Int. J. Mol. Sci..

[91] Sigala B., McKee C., Soeda J., Pazienza V., Morgan M., Lin C.I., Selden C., Borght S.V., Mazzoccoli G., Roskams T. (2013). Sympathetic Nervous System Catecholamines and Neuropeptide Y Neurotransmitters Are Upregulated in Human NAFLD and Modulate the Fibrogenic Function of Hepatic Stellate Cells. PLoS ONE.

[92] Kraft G., Vrba A., Scott M., Allen E., Edgerton D.S., Williams P.E., Vafai S.B., Azamian B.R., Cherrington A.D. (2019). Sympathetic Denervation of the Common Hepatic Artery Lessens Glucose Intolerance in the Fat- and Fructose-Fed Dog. Diabetes.

[93] Bruinstroop E., Eliveld J., Foppen E., Busker S., Ackermans M.T., Fliers E., Kalsbeek A. (2015). Hepatic denervation and dyslipidemia in obese Zucker (fa/fa) rats. Int. J. Obes..

[94] Hurr C., Simonyan H., Morgan D.A., Rahmouni K., Young C.N. (2019). Liver sympathetic denervation reverses obesity-induced hepatic steatosis. J. Physiol..

[95] Amir M., Yu M., He P., Srinivasan S. (2020). Hepatic Autonomic Nervous System and Neurotrophic Factors Regulate the Pathogenesis and Progression of Non-alcoholic Fatty Liver Disease. Front. Med..

[96] Kato H., Shimazu T. (1983). Effect of Autonomic Denervation on DNA Synthesis During Liver Regeneration After Partial Hepatectomy. Eur. J. Biochem..

[97] Li B., Wang H., Zhang Y., Liu Y., Zhou T., Zhou B., Zhang Y., Chen R., Xing J., He L. (2022). Current Perspectives of Neuroendocrine Regulation in Liver Fibrosis. Cells.

[98] Pimentel C.F.M.G., Salvadori R., Feldner A.C.D.C.A., Aguiar M.O.D., Gonzalez A.M., Branco G.R., Superbia M., Lai M., Mota D.D.O., Ferraz M.L.C.G. (2022). Autonomic dysfunction is common in liver cirrhosis and is associated with cardiac dysfunction and mortality: Prospective observational study. Sao Paulo Med. J..

[99] Colle I., Van Vlierberghe H., Troisi R., De Hemptinne B. (2004). Transplanted liver: Consequences of denervation for liver functions. Anat. Rec. Part A Discov. Mol. Cell. Evol. Biol..

[100] Koopman F.A., Stoof S.P., Straub R.H., Van Maanen M.A., Vervoordeldonk M.J., Tak P.P. (2011). Restoring the Balance of the Autonomic Nervous System as an Innovative Approach to the Treatment of Rheumatoid Arthritis. Mol. Med..

[101] Verlinden T.J.M., Van Dijk P., Hikspoors J., Herrler A., Lamers W.H., Köhler S.E. (2019). Innervation of the human spleen: A complete hilum-embedding approach. Brain Behav. Immun..

[102] Wei Y., Wang T., Liao L., Fan X., Chang L., Hashimoto K. (2022). Brain-spleen axis in health and diseases: A review and future perspective. Brain Res. Bull..

[103] Breit S., Kupferberg A., Rogler G., Hasler G. (2018). Vagus Nerve as Modulator of the Brain–Gut Axis in Psychiatric and Inflammatory Disorders. Front. Psychiatry.

[104] Lori A., Perrotta M., Lembo G., Carnevale D. (2017). The Spleen: A Hub Connecting Nervous and Immune Systems in Cardiovascular and Metabolic Diseases. Int. J. Mol. Sci..

[105] Moraes D.M.V.D., Gutierres A., Colleoni Neto R., Lindemann I.L., Rottenfusser R., Carlotto J.R.M. (2022). Anatomy of the splenic artery: What does the surgeon need to know?. Rev. Colégio Bras. Cir..

[106] Gupta I., Cassará A.M., Tarotin I., Donega M., Miranda J.A., Sokal D.M., Ouchouche S., Dopson W., Matteucci P., Neufeld E. (2020). Quantification of clinically applicable stimulation parameters for precision near-organ neuromodulation of human splenic nerves. Commun. Biol..

[107] Cleypool C.G.J., Lotgerink Bruinenberg D., Roeling T., Irwin E., Bleys R.L.A.W. (2021). Splenic artery loops: Potential splenic plexus stimulation sites for neuroimmunomodulatory-based anti-inflammatory therapy?. Clin. Anat..

[108] Bellinger D., Lorton D. (2018). Sympathetic Nerve Hyperactivity in the Spleen: Causal for Nonpathogenic-Driven Chronic Immune-Mediated Inflammatory Diseases (IMIDs)?. Int. J. Mol. Sci..

[109] Osborn J.W., Fink G.D. (2010). Region-specific changes in sympathetic nerve activity in angiotensin II-salt hypertension in the rat. Exp. Physiol..

[110] Sattler J., Tu J., Stoner S., Li J., Buttgereit F., Seibel M.J., Zhou H., Cooper M.S. (2018). Role of 11β-HSD type 1 in abnormal HPA axis activity during immune-mediated arthritis. Endocr. Connect..

[111] Donegà M., Fjordbakk C.T., Kirk J., Sokal D.M., Gupta I., Hunsberger G.E., Crawford A., Cook S., Viscasillas J., Stathopoulou T.-R. (2021). Human-relevant near-organ neuromodulation of the immune system via the splenic nerve. Proc. Natl. Acad. Sci. USA.

[112] Albaghdadi M., Garcia-Polite F., Zani B., Keating J., Melidone R., Spognardi A., Markham P., Tzafriri A. (2019). Splenic artery denervation: Target micro-anatomy, feasibility, and early preclinical experience. Transl. Res..

[113] Brudey C., Park J., Wiaderkiewicz J., Kobayashi I., Mellman T.A., Marvar P.J. (2015). Autonomic and inflammatory consequences of posttraumatic stress disorder and the link to cardiovascular disease. Am. J. Physiol.-Regul. Integr. Comp. Physiol..

[114] Elkhatib S.K., Moshfegh C.M., Watson G.F., Schwab A.D., Katsurada K., Patel K.P., Case A.J. (2021). Splenic denervation attenuates repeated social defeat stress-induced T-lymphocyte inflammation. Biol. Psychiatry Glob. Open Sci..

[115] Kummer W. (2011). Pulmonary Vascular Innervation and Its Role in Responses to Hypoxia: Size Matters!. Proc. Am. Thorac. Soc..

[116] Davies M.G., Miserlis D., Hart J.P. (2022). Current status of pulmonary artery denervation. Front. Cardiovasc. Med..

[117] Rothman A.M.K., Arnold N.D., Chang W., Watson O., Swift A.J., Condliffe R., Elliot C.A., Kiely D.G., Suvarna S.K., Gunn J. (2015). Pulmonary Artery Denervation Reduces Pulmonary Artery Pressure and Induces Histological Changes in an Acute Porcine Model of Pulmonary Hypertension. Circ. Cardiovasc. Interv..

[118] Chen S.-L., Zhang H., Xie D.-J., Zhang J., Zhou L., Rothman A.M.K., Stone G.W. (2015). Hemodynamic, functional, and clinical responses to pulmonary artery denervation in patients with pulmonary arterial hypertension of different causes: Phase II results from the Pulmonary Artery Denervation-1 study. Circ. Cardiovasc. Interv..

[119] Zhang H., Wei Y., Zhang C., Yang Z., Kan J., Gu H., Fan F., Gu H., Wang Q., Xie D. (2022). Pulmonary Artery Denervation for Pulmonary Arterial Hypertension. JACC Cardiovasc. Interv..

[120] Zhang H., Zhang J., Chen M., Xie D.-J., Kan J., Yu W., Li X.-B., Xu T., Gu Y., Dong J. (2019). Pulmonary Artery Denervation Significantly Increases 6-Min Walk Distance for Patients with Combined Pre- and Post-Capillary Pulmonary Hypertension Associated with Left Heart Failure: The PADN-5 Study. JACC Cardiovasc. Interv..

[121] Rothman A., Vachiery J.-L.E., Howard L.S., Mikhail G., Lang I.M., Jonas M., Kiely D.G., Abriel A., Lewis G.D., Rosenzweig E.B. (2020). Percutaneous Endovascular Ultrasound Pulmonary Artery Denervation for the Treatment of Pulmonary Arterial Hypertension: 12-Month Results of the Trophy 1 Study. Am. J. Respir. Crit. Care Med..

[122] Romanov A., Cherniavskiy A., Novikova N., Edemskiy A., Ponomarev D., Shabanov V., Losik D., Elesin D., Stenin I., Mikheenko I. (2020). Pulmonary Artery Denervation for Patients with Residual Pulmonary Hypertension After Pulmonary Endarterectomy. J. Am. Coll. Cardiol..

[123] Gold O.M.S., Bardsley E.N., Ponnampalam A.P., Pauza A.G., Paton J.F.R. (2022). Cellular basis of learning and memory in the carotid body. Front. Synaptic Neurosci..

[124] Badoer E. (2020). The Carotid Body a Common Denominator for Cardiovascular and Metabolic Dysfunction?. Front. Physiol..

[125] Platero-Luengo A., González-Granero S., Durán R., Díaz-Castro B., Piruat J.I., García-Verdugo J.M., Pardal R., López-Barneo J. (2014). An O_2_-sensitive glomus cell-stem cell synapse induces carotid body growth in chronic hypoxia. Cell.

[126] Tse A., Yan L., Lee A.K., Tse F.W. (2012). Autocrine and paracrine actions of ATP in rat carotid body. Can. J. Physiol. Pharmacol..

[127] Nurse C.A. (2014). Synaptic and paracrine mechanisms at carotid body arterial chemoreceptors. J. Physiol..

[128] Pardal R., Ortega-Sáenz P., Durán R., López-Barneo J. (2007). Glia-like stem cells sustain physiologic neurogenesis in the adult mammalian carotid body. Cell.

[129] Shin M.-K., Tang W.-Y., Amorim M.R., Sham J.S.-K., Polotsky V.Y. (2024). Carotid body denervation improves hyperglycemia in obese mice. J. Appl. Physiol..

[130] Marcus N.J., Del Rio R., Schultz E.P., Xia X.-H., Schultz H.D. (2014). Carotid body denervation improves autonomic and cardiac function and attenuates disordered breathing in congestive heart failure. J. Physiol..

[131] Search for: Other Terms: Renal Denervation|List Results|ClinicalTrials.gov. https://clinicaltrials.gov/search?term=renal%20denervation&viewType=Table#classicRedirect.

[132] Marchesini G., Forlani G., Cerrelli F., Manini R., Natale S., Baraldi L., Ermini G., Savorani G., Zocchi D., Melchionda N. (2004). WHO and ATPIII proposals for the definition of the metabolic syndrome in patients with Type 2 diabetes. Diabet. Med..

[133] John R.S., Dixon B., Hendrix J.M., Shienbaum R. (2024). Celiac Plexus Block. StatPearls.

[134] Quarti-Trevano F., Seravalle G., Dell’Oro R., Mancia G., Grassi G. (2021). Autonomic Cardiovascular Alterations in Chronic Kidney Disease: Effects of Dialysis, Kidney Transplantation, and Renal Denervation. Curr. Hypertens. Rep..

[135] Booth L.C., De Silva R.A.U., Pontes R.B., Yao S.T., Hood S.G., Lankadeva Y.R., Kosaka J., Eikelis N., Lambert G.W., Schlaich M.P. (2021). Renal, Cardiac, and Autonomic Effects of Catheter-Based Renal Denervation in Ovine Heart Failure. Hypertension.

[136] Brinkman D.J., Simon T., Ten Hove A.S., Zafeiropoulou K., Welting O., Van Hamersveld P.H.P., Willemze R.A., Yim A.Y.F.L., Verseijden C., Hakvoort T.B.M. (2022). Electrical stimulation of the splenic nerve bundle ameliorates dextran sulfate sodium-induced colitis in mice. J. Neuroinflamm..

[137] Pisano G., Fracanzani A.L., Caccamo L., Donato M.F., Fargion S. (2016). Cardiovascular risk after orthotopic liver transplantation, a review of the literature and preliminary results of a prospective study. World J. Gastroenterol..

[138] Jordan J., Tank J. (2020). How Sympathetic Is Sympathetic Enough?. Hypertension.

[139] Kantauskaite M., Vonend O., Yakoub M., Heilmann P., Maifeld A., Minko P., Schimmöller L., Antoch G., Müller D.N., Schmidt C. (2023). The Effect of Renal Denervation on T Cells in Patients with Resistant Hypertension. Int. J. Mol. Sci..

[140] Oliveras A., Armario P., Sans L., Clarà A., Vázquez S., Molina L., Pareja J., De La Sierra A., Pascual J. (2018). Organ damage changes in patients with resistant hypertension randomized to renal denervation or spironolactone: The DENERVHTA (Denervación en Hipertensión Arterial) study. J. Clin. Hypertens..

[141] Verloop W.L., Vink E.E., Spiering W., Blankestijn P.J., Doevendans P.A., Bots M.L., Vonken E., Voskuil M., Leiner T. (2015). Effects of renal denervation on end organ damage in hypertensive patients. Eur. J. Prev. Cardiol..

[142] Kiuchi M.G., Schlaich M.P., Chen S., Villacorta H., Ho J.K., Carnagarin R., Matthews V.B., Lugon J.R. (2019). Relevance of Targeting the Distal Renal Artery and Branches with Radiofrequency Renal Denervation Approaches—A Secondary Analysis from a Hypertensive CKD Patient Cohort. J. Clin. Med..

[143] Choi K.H., Choi S.-H. (2021). Current Status and Future Perspectives of Renal Denervation. Korean Circ. J..

[144] Roubsanthisuk W., Kunanon S., Chattranukulchai P., Panchavinnin P., Wongpraparut N., Chaipromprasit J., Pienvichitr P., Ayudhya R.K.N., Sukonthasarn A., on behalf of Thai Hypertension Society (2023). 2022 Renal denervation therapy for the treatment of hypertension: A statement from the Thai Hypertension Society. Hypertens. Res..

[145] Wolf M., Hubbard B., Sakaoka A., Rousselle S., Tellez A., Jiang X., Kario K., Hohl M., Böhm M., Mahfoud F. (2018). Procedural and anatomical predictors of renal denervation efficacy using two radiofrequency renal denervation catheters in a porcine model. J. Hypertens..

[146] Ettehad D., Emdin C.A., Kiran A., Anderson S.G., Callender T., Emberson J., Chalmers J., Rodgers A., Rahimi K. (2016). Blood pressure lowering for prevention of cardiovascular disease and death: A systematic review and meta-analysis. Lancet.

